# Barriers and facilitators to interhospital transfer of acute pulmonary embolism: An inductive qualitative analysis

**DOI:** 10.3389/fmed.2023.1080342

**Published:** 2023-03-01

**Authors:** Jacob DeBerry, Parth Rali, Michael McDaniel, Christopher Kabrhel, Rachel Rosovsky, Roman Melamed, Oren Friedman, Jean M. Elwing, Vijay Balasubramanian, Sandeep Sahay, Eduardo Bossone, Mary Jo S. Farmer, Andrew J. P. Klein, Megan E. Hamm, Charles B. Ross, Belinda N. Rivera-Lebron

**Affiliations:** ^1^Department of Medicine, University of Pittsburgh, Pittsburgh, PA, United States; ^2^Department of Medicine, Temple University, Philadelphia, PA, United States; ^3^Department of Medicine, Emory University, Atlanta, GA, United States; ^4^Massachusetts General Hospital and Harvard Medical School, Boston, MA, United States; ^5^Abbott Northwestern Hospital, Minneapolis, MN, United States; ^6^Cedars-Sinai Medical Center, Los Angeles, CA, United States; ^7^College of Medicine, University of Cincinnati, Cincinnati, OH, United States; ^8^School of Medicine, University of California San Francisco, San Francisco, CA, United States; ^9^Houston Methodist Hospital, Houston, TX, United States; ^10^A. Cardarelli Hospital, Naples, Italy; ^11^Baystate Medical Center, University of Massachusetts Baystate, Springfield, MA, United States; ^12^Piedmont Atlanta Hospital, Atlanta, GA, United States

**Keywords:** pulmonary embolism, interhospital transfer, pulmonary embolism response team, catheter–directed thrombolysis, surgical embolectomy

## Abstract

**Background:**

Interhospital transfer (IHT) of patients with acute life-threatening pulmonary embolism (PE) is necessary to facilitate specialized care and access to advanced therapies. Our goal was to understand what barriers and facilitators may exist during this transfer process from the perspective of both receiving and referring physicians.

**Methods:**

This qualitative descriptive study explored physician experience taking care of patients with life threatening PE. Subject matter expert physicians across several different specialties from academic and community United States hospitals participated in qualitative semi-structured interviews. Interview transcripts were subsequently analyzed using inductive qualitative description approach.

**Results:**

Four major themes were identified as barriers that impede IHT among patients with life threatening PE. *Inefficient communication* which mainly pertained to difficulty when multiple points of contact were required to complete a transfer. *Subjectivity in the indication for transfer* which highlighted the importance of physicians understanding how to use standardized risk stratification tools and to properly triage these patients. *Delays in data acquisition* were identified in regards to both obtaining clinical information and imaging in a timely fashion. *Operation barriers* which included difficulty finding available beds for transfer and poor weather conditions inhibiting transportation. In contrast, two main facilitators to transfer were identified: *good communication and reliance on colleagues* and *dedicated team for transferring and treating PE patients*.

**Conclusion:**

The most prominent themes identified as barriers to IHT for patients with acute life-threatening PE were: (1) inefficient communication, (2) subjectivity in the indication for transfer, (3) delays in data acquisition (imaging or clinical), and (4) operational barriers. Themes identified as facilitators that enable the transfer of patients were: (1) good communication and (2) a dedicated transfer team. The themes presented in our study are useful in identifying opportunities to optimize the IHT of patients with acute PE and improve patient care. These opportunities include instituting educational programs, streamlining the transfer process, and formulating a consensus statement to serve as a guideline regarding IHT of patients with acute PE.

## Introduction

Acute pulmonary embolism (PE) is the third leading cause of cardiovascular death in the United States (1, 2). Timely triage, diagnosis, risk stratification and treatment of an acute PE are critical. With recent advances, the treatment of acute PE has become complex, ranging from anticoagulation alone to catheter-based interventions, and surgical thrombectomy with mechanical support (3–5). Given the breadth of advanced therapies available, in certain settings, patients may require interhospital transfer (IHT) to a tertiary care facility to have access to multidisciplinary pulmonary embolism response teams (PERT) (6).

The most recent European Guidelines for management of acute PE recognize the value of PERTs in the management of intermediate and high-risk PE patients (4). In some centers, PERT implementation has led to increased number of advanced therapies, without an increase in major bleeding (7). Additionally, numerous studies have demonstrated that having a PERT decreases length of stay, costs and even mortality (8–13).

Transferring patients between institutions, to facilitate advanced care may be necessary to achieve optimal clinical outcomes and salvage of life, but it also represents a period of heightened vulnerability for patients as well as physicians responsible for their care. IHT has been extensively studied, for numerous acute medical conditions such as trauma, acute ST-elevation myocardial infarction and stroke (14–16). However, the process of IHT for PE is less well understood. The aim of this study was to investigate barriers and facilitators of transferring patients with acute PE amongst physicians who are responsible for both the transferring and receiving of patients, with the eventual goal of learning how to better streamline the process and improve patient safety.

## Methods

A writing group was established by members of the Clinical Protocols Committee of the PERT Consortium™. This study was approved by the University of Pittsburgh Institutional Review Board (Study #20070380). The consolidated criteria for reporting qualitative studies (COREQ) guidelines was reviewed and adhered to, where applicable (17).

### Recruitment

**Figure 1:**
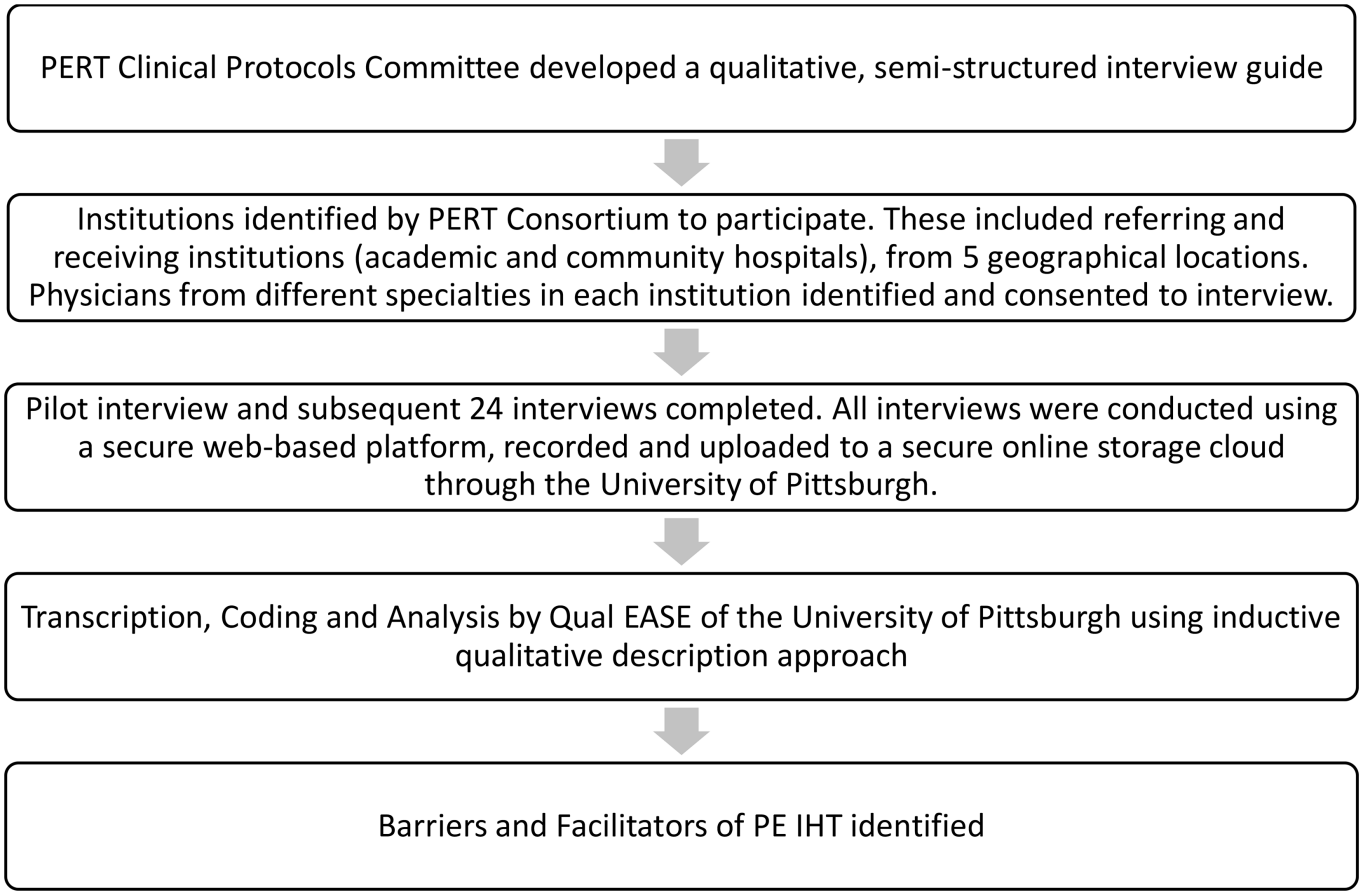
Methods flow chart. PERT, pulmonary embolism response team. Qual EASE, qualitative, evaluation, and stakeholder engagement research core of the Center for Research on Healthcare’s Data Center at the University of Pittsburgh. IHT, interhospital transfer. PE, pulmonary embolism.

Twenty-nine subject matter expert physicians who typically refer (“referring physicians”) and physicians who typically receive (“receiving physicians”) PE patients were identified by members from the Clinical Protocols Committee of the PERT Consortium™ (Figure 1). Twenty-five of the identified expert physicians participated in the study, while four of the physicians were omitted because there was no email correspondence back from them. The physicians who were recruited and interviewed were colleagues of members of The PERT Consortium™, but not active members. Consortium members were asked to identify participants from different specialists (pulmonary, critical care, anesthesiology, and emergency medicine), different types of hospitals (academic and community) and different geographic areas in the United States. An invitation letter was sent to each physician *via* email that explained the goal of the study and included the study consent form. Twenty-four of the participants were provided a $250 honorarium to compensate them for their time, while one participant declined the honorarium citing that it could be seen as a conflict of interest. Funding was provided by a grant from the Boston Scientific Corporation to the PERT Consortium™.

### Interviews

A semi-structured interview guide was created by members of the Clinical Protocols Committee of The PERT Consortium™ for the referring physicians and for the receiving physicians (Table 1). It included questions on the process of transferring or receiving patients with life threatening PE, its barriers, and facilitators. Most questions had an open answer. Seven iterations were revised before the final interview guide was agreed upon.

**Table 1:** Semi-structured interview guide.

**Referring hospital interview guide** Let us start by having you briefly describe what you do What is your specialty? How long have you been practicing in this specialty? What is your role and experiences with patients with PE? How many PE patients do you evaluate a year? Approximately What percent of them get transferred a tertiary-care hospital? Approximately Tell me about navigating the process of transferring patients with life-threatening PE from your institution to a tertiary-care hospital Do you find transfer process easy or hard? Are there single points of contact or multiple steps required to complete a transfer? Generally, what is the estimated time from acceptance to actual transfer of patient with PE? What aspects of transferring patients with life-threatening PE to a tertiary-care hospital have been well? (free response) What aspects of transferring patients with life-threatening PE have been challenging? (free response) The following questions address issues related to transfer Have you ever had a patient decompensate or die during the transfer process? What percent of patients with PE deteriorate while awaiting transfer, less than 50% or greater than 50%? What do you do once a patient is accepted and then decompensates in your care? Have you experienced any difficulty with identifying an appropriate patient to transfer? Prior to transfer, do you call the desired accepting center first for assistance, make the decision to transfer on your own, or consult your own subspecialty physicians for assistance? Is discussing contingency plans part of normal transfer process? What specific information do you think tertiary care centers would like to know prior to accepting a transfer for a patient with a life-threatening PE? (free response) Regarding information needed for transfer Is there any difficulty at your hospital obtaining this information in a timely manner prior to transfer? Do you have systemic ways of transferring imaging to accepting hospitals at all the times? Are associated data and imaging always sent, even during nights and weekends? Difficulty with transportation? Do you have any ideas about how to improve these transitions of care? (free response) Lastly, is there anything else you could share with me that might help me better understand these transitions of care? (free response)
**Receiving hospital interview guide** Let us start by having you briefly describe what you do What is your specialty? How long have you been practicing in this specialty? What is your role and experiences with patients with pulmonary embolism? How many PE patients do you evaluate a year? Approximately Tell me about navigating the process of accepting the care of patients with life-threatening PE transferring into your facility (free response) What aspects of care transitions of patients with life-threatening PE have been going smoothly? (free response) What aspects of care transitions of patients with life-threatening PE have been challenging? (free response) What information do you specifically want to know from a facility requesting to transfer a patient with a life-threatening PE? (free response) The following questions address issues related to transfer Do you know, approximately how many PE transfers does your hospital accept a month? Usually, what is the average time from acceptance to arrival? What percent of patient’s acutely deteriorate upon arrival? What percent of patients are admitted directly to ICU level care vs. non-ICU level of care? Do you have ideas about how to improve these transitions of care? (free response) Is there anything else you could share with me that might help me better understand these transitions of care? (free response)

A pilot interview was conducted by a study investigator (JD), a male internal medicine physician. JD was familiar with basic interview techniques, but did not have formal training with semi-structured interviews. The pilot interview was completed with one receiving physician, and reviewed by another study investigator (BRL) who is a female pulmonary and critical physician, to ensure all questions were easily understood and relevant. After the pilot interview, no additional changes were made to the study questions. All interviews were conducted remotely with video interface by JD using a secure web-based platform and were recorded with password protection. Once completed, the interviews were uploaded to a secure online storage service through the University of Pittsburgh. The interviewer, JD, had no prior relationships with the physicians that were interviewed.

### Data collection and analysis

The interviews were evaluated by the Qualitative, Evaluation, and Stakeholder Engagement Research Core of the Center for Research on Healthcare’s Data Center at the University of Pittsburgh (Qual EASE) for analysis. All audio files were transcribed verbatim with identifying details redacted.

Analysis followed an inductive qualitative description approach (18, 19), a common qualitative approach in medical studies, in which the analysts focused on describing the viewpoints of the interviewees as closely as possible without abstracting to the level of social theory. Interview analysis was completed by a team of trained qualitative analysts at the Qual EASE within the University of Pittsburgh. An experienced Qual EASE analyst created a codebook inductively from the content of the interviews and categorized participant responses to “barriers” or “facilitators” of PE patient care during the IHT process. This analyst and a second analyst then practiced using the codebook on 4 transcripts, meeting afterwards to adjudicate coding and refine the codebook as necessary. Subsequently, they then independently coded 10 transcripts for the purposes of measuring intercoder reliability using Cohen’s kappa scores for each code in the codebook. The average kappa score was 0.75, indicating substantial agreement (20). The two coders adjudicated all coding disagreements to full agreement, and then the primary coder coded the remaining transcripts independently. The resulting coding was used to conduct both conventional content (21) and thematic (22, 23) analyses of the results. The content analysis summarized was discussed in the interviews and served as step one of the six-phase guide to thematic analysis by Braun and Clarke (14), which was followed in order to conduct a thematic analysis: (1) familiarization with data, (2) generation of initial code, (3) search for themes, (4) review of themes, (5) definition and names of themes, and (6) production of the report. Final analysis consisted of sorting coded barriers and facilitators to transfer in the text into themes, which were then shared with the broader study team for corroboration by the interviewer, and to allow the study team to give the coders and analysts feedback that helped to better flesh out resulting themes.

## Results

Twenty-five interviews were conducted between September 21, 2020 and December 11, 2020 (Table 2). Themes are described in depth below, with supporting quotes presented both in-text and in Table 3. Quotes in the table are cited with a quote number, e.g., (Quote 1).

**Table 2:** Referring and receiving institutions and specialties that participated in semi-structured interviews.

**Receiving institutions**		
**Geographical region**	**Institution**	**Specialty** (n = number of physicians interviewed)
West Coast	Cedars Sinai	Pulmonary and critical care (1)
Midwest	Abbott Northwestern Hospital	Pulmonary and critical care (1) Anesthesiology and critical care (1)
Ohio Valley	UPMC	Pulmonary and critical care (1) Emergency medicine (1)
South	Piedmont Atlanta	Pulmonary and critical care (1)
East	Harvard Affiliated Hospital	Emergency medicine (2)
**Referring institutions**		
West Coast	Cedars Sinai Marina del Rey	Emergency medicine (3)
Midwest	Abbott Northwestern Affiliated Hospital St. Francis Regional Medical Center	Emergency medicine (3)
Ohio Valley	UPMC Affiliated Hospitals	Emergency medicine (3)
South	Piedmont Affiliated Hospitals Southern Regional Medical Center	Emergency medicine (3) Pulmonary and critical care (1)
East	Cooley Dickinson Health Care Martha’s Vineyard Hospital Salem Hospital	Emergency medicine (4)

**Table 3:** Quotes representing barriers and facilitators to interhospital transfer of pulmonary embolism (PE) patients.

**Quote #**	**Quote**
**Barriers to interhospital transfer PE patients**	
Inefficient communication	
1	*“It’s usually quite cumbersome, and that becomes especially so if we are dealing with more than one hospital*.” (EM 6 – referring)
2	*“If I can see all of the objective data, again, I feel very, uh, much more comfortable with our plan than if I cannot see that.”* (PCCM 1–receiving)
Subjectivity in indication for transfer	
3	“*So, the patient in my opinion met criteria for giving tPA. I called the PERT, I spoke with one of the physicians there. I cannot remember if it was resident or an attending. We kind of went through some of the criteria. He thought the patient did not meet criteria for tPA. And then it was a question of, you know, okay, should this patient be transferred or not? We kind of had a discussion about that. I remember talking to the hospitalist at our institution who felt the patient should be transferred. The PERT team thought that we could manage the patient at our facility. I felt pretty strongly about giving the tPA given the clinical picture and the criteria that she was meeting. So, I sort of pushed for the tPA even though the PERT team doc kinda did not feel strongly about it, wasn’t really recommending it, but said, you know, if I want to give it I can give it.”* (EM 7 – referring)
4	*“Something that’s frustrating is that it’s sometimes a lot of subjective information [that] is used in the decision-making process. We do not always use strict objective criteria, so it may depend on who you get on the other line, you know, in other words… we have had patients who have had borderline troponin, borderline echo findings, BNP. They may not have all the-the kind of the objective criteria that we at least look for, which are elevated troponin, elevated BNP, evidence of right heart strain, hypoxia, you know. They may have one or two of those factors and then age may play a role, and then clot burden is, you know, subjective based on a review of the CT scan. So, you know, I’ve had instances where I feel like I’ve had patients with fairly similar characteristics; one gets accepted, one does not.”* (PCCM 2 – referring)
Delays in data acquisition	
5	“*What we do have difficulty sometimes, depending on the staffing of the referring hospital, is that they sometimes have difficulty getting an echo, STAT, during off-hours*.” (PCCM 3 – receiving)
6	*“[After imaging], the other half is getting the specialists to see the patient and see the imaging and make that recommendation.”* (EM 2 – receiving)
7	*“Occasionally, um, it can be, uh, there may be a slight delay in obtaining the echo, the STAT echo, simply because of the volume of patients we have.”* (PCCM 2 – referring)
8	“*They do not understand the nature of emergency medicine that there are many patients that you do not have the luxury of waiting 45 min to an hour to get labs back, to do testing, and that these patients need to be tested quickly and immediately.”* (EM 8 – referring)
Operational barriers	
9	*“At the end of the day, none of them are obligated to accept, no matter what the situation is. And so, we have had situations where we have had very sick people who have had delayed transport.”* (EM 3 – referring)
10	*“Where I work now, it’s a whole different ballgame, because there’s no land route. So, ambulances need to get on a ferry in order to get the patient off. So, our primary access for transferring crucially ill patients is MedFlight and, depending on the weather, if the helicopters aren’t running and you really have a critical patient, we have to send them off by Coast Guard.”* (EM 5–referring)
11	*“Sometimes insurances do not agree that this is a high level of care requirement. […] Typically, as an emergency physician, I’m still in charge of making that determination. It just comes with a lot of challenges to a patient [whose] bill is declined or denied by their insurance carrier. But sometimes that will make the transfer more challenging because there’s other physicians involved who will not authorize it, and it makes it more challenging. Especially in cases where it’s unclear that the patient may need a procedure but should probably be at a higher level of care institution in case they deteriorate.”* (EM 9–referring)
12	*“Sometimes you just have to try to get the insurance company to authorize the ambulance because the patient does not want to get stuck with a bill, and then the insurance company could say, well, we do not want to offer it to transfer despite it being a higher level of care.”* (EM 10 – referring)
**Facilitators to interhospital transfer PE patients**	
Good communication and reliance on colleagues	
13	*“I think our ability to initiate either tPA or heparin is—We have pharmacists in our staff, our hospital, who are very competent and are very helpful in initiating meds. So, I think that part is very good.”* (EM 4 – referring)
14	*“[Because of] exceptional EMS providers and flight crew providers, the transfers, from a critical care standpoint tend to go pretty well. They’re very good at care of patients en-route. They often work to stabilize patients who they pick up, even prior to transporting them. So generally, those aspects of transfer are some of the best around.”* (EM 11 – receiving)
Dedicated transfer and treatment teams (i.e., PERT)	
15	*“We did not always have these PERT teams. Before, we kind of had to have a more ad hoc approach to how we care for these patients. Now we have clear definitions of what a massive and submassive PE, and then clear, accountable consultants who get notified, and then this kind of path to the CCU for those massive PEs. It’s more clearly defined, and so, it – you know, trying to find an ICU bed for these patients is less difficult than it used to be.”* (EM 2 – receiving)

BNP, brain natriuretic peptide; CCU, cardiac care unit; CT scan, computed tomography scan; ICU, intensive care unit; EM, emergency medicine physician; EMS, emergency medical services; PCCM, pulmonary and critical care medicine physician; PE, pulmonary embolism; PERT, pulmonary embolism response team; tPA, tissue plasminogen activator.

### Barriers to interhospital transfer of pulmonary embolism patients

Several barriers to a smooth transfer process for PE patients were identified. Barriers to transfer process include: (1) inefficient communication, (2) subjectivity in the indication for transfer, (3) delays in data acquisition (imaging or clinical), and (4) operational barriers, including lack of available beds and poor weather conditions inhibiting transportation, and patient desires and/or informed decision making, to avoid transfer for personal or financial reasons.

#### Inefficient communication

Inefficient communication among physicians causes perceived delays transferring PE patients. For initiating transfers, referring physicians struggle in their efforts to make initial contact with receiving centers. Having no single point of contact may cause time delays (Quote 1). For referring physicians not part of a larger network, it can take multiple phone calls to different facilities to find one that will accept a transfer. Furthermore, lack of centralized call systems was reported as a contributor to physicians not being able to efficiently initiate a transfer.

Physicians also reported challenges when they do not share the same medical record system, radiology images or patient data. The inability to share images and data in real time delays care. Although images can be sent with the patient on a disk, this approach still represents a delay for accepting physicians. The more information receiving physicians have prior to patient arrival, the sooner they can prepare for the patient. Also, receiving physicians can make better clinical judgments when they can evaluate all the patient’s data while talking on the phone with the referring physician (Quote 2).

#### Subjectivity in the indication for transfer

The subjectivity involved in identifying and triaging PE patients between referring and receiving physicians creates challenges on both ends, in that physicians can struggle to decide if a patient needs to be transferred. Participants report that it is not always clear which patients need to be transferred. This lack of clarity can lead to disagreements amongst physicians. For example, a referring physician may risk stratify a patient using imaging alone but receiving physicians might refer to imaging and biomarkers or other test results that the referring physician may not have access to or may not routinely use in their clinical practice (Quote 3). Referring physicians find it frustrating when they want to transfer a patient and the transfer is not approved by the accepting facility (Quote 4).

The referring physician may feel that their center lacks the proper treatment, level of care or expertise for the patient, or may personally feel uncomfortable starting treatments beyond anticoagulation alone. “*It’s hard to make that decision to [either use heparin or tissue plasminogen activator (tPA) and then start heparin after the tPA after an undisclosed period of time.” [Emergency medicine (EM) physician 1]* Sometimes referring physicians are not comfortable administering tPA and will transfer a patient without doing so. This deferment can delay patient care and affect patient outcomes.

Additionally, managing patients that decompensate prior to or during transfer is a challenge. Referring physicians may not adequately stabilize a patient prior to transfer or the transport team may need to manage decompensating patients *in route*. Additionally, waiting for the appropriate emergency medical service crew (ground or air, personnel, equipment and monitoring required during transfer, etc.) to transfer PE patients may cause delays in patients receiving the care they need. Patients who are too unstable also might not qualify for transfer.

#### Delays in data acquisition

Referring physicians described difficulty reaching other individuals needed to perform imaging, particularly off-hours (Quote 5). Delays in imaging result in delays in patient care (Quote 6). High patient volume can also cause delays in obtaining imaging (Quote 7).

Furthermore, delays may be caused by lack of understanding of the urgency of the situation by those involved in imaging. Referring physicians found a lack of urgency from radiologists and computed tomography (CT) technicians. Radiologists want documentation of the medical risks, such as acute kidney injury patients may experience, before approving scans (Quote 8). On the receiving end, physicians encounter a lack of urgency from providers for hemodynamically unstable PE patients. It is “*frustrating from an emergency medicine perspective” [EM 2]* that these patients must wait to be triaged before they are evaluated by a PE consultant.

#### Operational barriers

Several structural and situational barriers interfere with the transfer process. A lack of bed availability impacts both accepting and referring physicians. If the referring physician’s facility does not have an open bed for a PE patient, they must transfer that patient. Receiving facilities are not able to accept patients if there are no beds available in their institution and may subsequently, deny a transfer based on this circumstance alone (Quote 9).

Further delays can come from poor weather for transport, which can prevent helicopters from flying. Moreover, poor weather conditions particularly impact hospitals in unique locations, such as islands or remote locations, in that both ground and air transportation may be hindered (Quote 10).

Additionally, physicians encounter barriers to transfer from their patients. Patients worry about staying local and close to their families as well as covering the cost of transfer. These concerns need to be mediated by the referring physician before they initiate transfer. The time it takes to assure patients that transferring them is best for their care can cause significant delays, during which time they may not be getting the necessary care they need. Further, getting the patient’s insurance to authorize and cover the cost for the transfer and higher level of care may delay the patients’ getting to the accepting site (Quotes 11 and 12).

### Facilitators to interhospital transfer of pulmonary embolism patients

Facilitators of the transfer process are often the inverse of the barriers. Two main facilitators to transfer were identified: (1) Good communication and reliance on colleagues and, (2) The use of a dedicated team for transferring and treating PE patients (i.e., PERT) helps facilitate better communication between physicians and identify appropriate patients for transfer. Each of these will be discussed in turn.

#### Good communication and reliance on colleagues

Physicians find that centralized call centers are useful in facilitating transfers, because centralized call centers provide a single point of contact at receiving facilities with staff available at all times to quickly discuss and initiate a transfer. As one participant put it: “*So, for PE, [PERT] is generally one-stop shopping.*” *[EM 3]* A centralized call center is “*much more streamlined*” *[EM 4]* than having to call multiple facilities to find one that will receive a transfer.

Additionally, sharing a medical record system is “s*eamless in terms of the continuity. All our notes, labs, x-rays, everything is available to that institution.” [EM 5]* Physicians are able to access patient information while on the phone with the referring physician and communicate in real time what the treatment plan would be. Receiving physicians also can view patient data prior to the patient’s arrival to determine and coordinate the treatment plan before the patient arrives. This is particularly useful for those patients in whom a catheter-based or surgical intervention is anticipated.

Physicians also praised the personnel support they received during the transfer process. Pharmacists in the referring emergency department help initiate necessary medications (Quote 13). Transport teams were praised for their quick response times and the care they provide patients *in route* (Quote 14).

#### Dedicated transfer and treatment teams (i.e., pulmonary embolism response teams)

PERT was praised as helping to facilitate the transfer process. Participants describe that PERT defines clear treatment protocols that identify massive (or high risk) versus submassive (or intermediate risk) PE. These protocols help physicians determine where patients go once transferred, e.g., the intensive care unit or a lower level of care, and if advanced therapies are indicated. This accessibility allows physicians who are unsure what to do with a patient to consult PERT for next steps (Quote 15).

## Discussion

The transfer of critically ill patients can be complex but is often necessary. To our knowledge, this is the first qualitative study to examine the perceived barriers of IHT in patients with an acute PE. Previous qualitative research has examined barriers and perceived risk related to IHT for patients with life threatening medical condition other than PE. Overarching themes identified included inadequate communication, gaps in clinical practice, and lack of structure guiding the IHT process as barriers to care. Other studies have identified transfer delays, failure to transmit records, and setting unrealistic expectations as threats to the safety of patients requiring IHT (24).

In a recent review (6), our group described topics that should be included in the discussions between transferring and receiving physicians. Topics included patient presentation, current hemodynamic status, anticoagulation, relevant medical history, code status, laboratory and imaging results, and contingencies for management in case of deterioration. Many of these topics were evident in the interviews conducted for the current study. Education for transferring physicians on an “optimal transfer process” including items such as “checklists for discussion” may smooth the transfer process and represents another opportunity in our ongoing work to further improve this process.

Studies demonstrate that patients have improved 30 day survival when transferred to a high volume center with physicians who are more experienced with treating acute PE (25). Thus, it is important that the IHT process be as seamless as possible.

It appears that lack of communication can be generalized across all four barriers identified in the transfer process. Inefficient communication may include poor physician-to-physician communication, poor communication with call centers, and lack of access to electronic medical records (EMR), all of which hinder IHT. Lack of access to medical information presents a difficult challenge when an accepting physician is faced with the decision to accept a patient with an acute PE, particularly without being able to review relevant imaging and laboratory studies. Importantly, communication barriers also exist among patients and physicians which may be exacerbated when patients sometimes are resistant to the idea of transfer for reasons such as cost or distance from their home or family members.

In contrast, facilitators to IHT identified from our interviews translate to utilizing a central transfer center, discussing the case with a dedicated PERT member, and sharing the electronic medical record system. All of these factors, facilitate communication, and expedite the transfer process with the intent of improving patient care.

### Limitations

While a potential limitation to this study is the number of interviews conducted (25), physicians from different geographical locations and specialties across the United States were recruited, which diversified our understanding of the processes of transfer, EMRs, and patient population. Another limitation is that the physicians interviewed were either from institutions with a PERT (“receiving physicians”) or referring to an institution with a PERT (“accepting physicians”), their experiences with managing acute PE may not be representative of all providers. A single pilot interview was performed; however, no changes were deemed necessary after this pilot interview, and the subsequent interviews that were conducted yielded consistent codable data. Lastly, while we identified these broad themes related to barriers and facilitators to IHT, they may not all pertain to all hospital systems. It should be noted that having a clear understanding of a specific hospital system is needed prior to considering which barrier or facilitator is relevant.

## Conclusion

By identifying these broad themes, the current study can be used for process improvement and should stimulate further investigation into the safety of patient transfers. These activities may include educational programs for transferring facilities on risk stratification and indications for advanced therapies, which may then help identify which patients are most appropriate for transfer. Furthermore, the development of a checklist to standardize the course of transferring and receiving acute PE patients may help assure adequate information is readily available to the receiving institution, similar the one already developed by The PERT Consortium™ (6). Ultimately, formulating consensus and best practices to serve as a guideline in IHT may improve the care of patients with life-threatening acute PE.

## Data availability statement

The raw data supporting the conclusions of this article will be made available by the authors, without undue reservation.

## Ethics statement

The studies involving human participants were reviewed and approved by University of Pittsburgh Institutional Review Board (Study #20070380). The patients/participants provided their written informed consent to participate in this study.

## Author contributions

JD, BR-L, CK, PR, MM, RR, RM, OF, MH, AK, and CR were responsible for content development. JD, BR-L, CK, PR, MM, RR, RM, MH, AK, and CR wrote the first draft. JE, VB, SS, EB, MF, JD, BR-L, CK, PR, MM, RR, RM, OF, MH, AK, and CR contributed to concept, critical revision, and final approval of the manuscript. All authors contributed to the article and approved the submitted version.

## Funding

Funding was provided by a grant from the Boston Scientific Corporation to the PERT Consortium™.

## Conflict of interest

CK is consultant for BMS and Abbott, and provided by a grant from Grifols and Diagnostica Stago. RR is consultant for Abbott, BMS, Dova, Janseen, Inari Medical, and Penumbra, and provided by a grant from Janssen and BMS. OF is consultant for Inari Medical. BR-L is consultant for Inari Medical and Johnson & Johnson, and provided by a grant from Johnson & Johnson.

The remaining authors declare that the research was conducted in the absence of any commercial or financial relationships that could be construed as a potential conflict of interest.

## Publisher’s note

All claims expressed in this article are solely those of the authors and do not necessarily represent those of their affiliated organizations, or those of the publisher, the editors and the reviewers. Any product that may be evaluated in this article, or claim that may be made by its manufacturer, is not guaranteed or endorsed by the publisher.
